# Exploring the Key Genes and Pathways in the Formation of Corneal Scar Using Bioinformatics Analysis

**DOI:** 10.1155/2020/6247489

**Published:** 2020-01-18

**Authors:** Binfeng Liu, Ang Li, Hongbo Wang, Jialin Wang, Gongwei Zhai, Haohao Ma, Shiqing Feng, Liyun Liu, Yanzheng Gao

**Affiliations:** ^1^Zhengzhou University People's Hospital, Henan Provincial People's Hospital, Henan, China; ^2^Department of Orthopaedics, Henan Provincial People's Hospital, People's Hospital of Zhengzhou University, School of Clinical Medicine, Henan University, Zhengzhou, Henan, China; ^3^Henan University People's Hospital, Henan Provincial People's Hospital, Henan, China; ^4^Department of Orthopedics, Tianjin Medical University General Hospital, China; ^5^Luoyang Orthopedic Hospital of Henan Province, Orthopedic Hospital of Henan Province, Zhengzhou, China

## Abstract

The Corneal wound healing results in the formation of opaque corneal scar. In fact, millions of people around the world suffer from corneal scars, leading to loss of vision. This study aimed to identify the key changes of gene expression in the formation of opaque corneal scar and provided potential biomarker candidates for clinical treatment and drug target discovery. We downloaded Gene expression dataset GSE6676 from NCBI-GEO, and analyzed the Differentially Expressed Genes (DEGs), Gene Ontology (GO), Kyoto Encyclopedia of Genes and Genomes (KEGG) enrichment pathway analyses, and protein-protein interaction (PPI) network. A total of 1377 differentially expressed genes were identified and the result of Functional enrichment analysis, Kyoto Encyclopedia of Genes and Genomes (KEGG) identification and protein-protein interaction (PPI) networks were performed. In total, 7 hub genes IL6 (interleukin-6), MMP9 (matrix metallopeptidase 9), CXCL10 (C-X-C motif chemokine ligand 10), MAPK8 (mitogen-activated protein kinase 8), TLR4 (toll-like receptor 4), HGF (hepatocyte growth factor), EDN1 (endothelin 1) were selected. In conclusion, the DEGS, Hub genes and signal pathways identified in this study can help us understand the molecular mechanism of corneal scar formation and provide candidate targets for the diagnosis and treatment of corneal scar.

## 1. Introduction

The cornea is a hard, transparent tissue through which light enters the eyes first. It is transparent, without blood vessels, and has many sensory nerve endings. Serving as a barrier to debris and infection as well as two-thirds of the refractive power of the eye, the cornea is imperative to proper vision [1]. Cornea is part of the eye exposed to the outer environment and thus most likely to sustain damage due to various insults, such as trauma, inflammation, infection, and so on [2]. The corneal wound healing results in the formation of opaque corneal scar. In fact, millions of people around the world have corneal scars that cause vision loss [3]. Corneal transplantation is the traditional treatment for clinically significant corneal opacities. Regenerative medicine for the cornea represents a novel treatment strategy for patients with corneal diseases. Stem cell-based therapies represent a novel strategy that in some instances (using stromal stem cells) may substitute conventional corneal transplantation [1, 4]. The formation of corneal scar is a complex process: its mechanism and potential genetic control are not fully understood. It involves the synthesis of various growth factors, cytokines, and proteases produced by epithelial cells, stromal keratocytes, inflammatory cells, and lacrimal gland cells, [5]. Therefore, it is very important to understand the precise molecular mechanism of corneal scar formation and formulate effective diagnosis and treatment strategies. Gene chip or gene mapping is a gene detection technology that has been used for more than ten years. Gene chip can quickly detect all genes in the expression information of the same sample at the same time point. It is especially suitable for screening differentially expressed genes, [6]. We can use microarray technology and bioinformatics analysis to screen genetic alterations at the genome level [7]. Using microarray technology and bioinformatics analysis, we can accurately determine the differential expression level of genes, thus providing an effective method for large-scale gene expression research [8, 9]. In previous articles, some potential differentially expressed genes, hub genes, and signal pathways related to corneal scar formation have not been identified, which may help us understand the molecular mechanism of corneal scar formation and provide candidate targets for the treatment of corneal scar. In addition, there are no researches about differential gene analysis, Gene Ontology (GO), Kyoto Encyclopedia of Genes and Genomes (KEGG) enrichment pathway analyses, and protein-protein interaction (PPI) network analyses of corneal scar using the dataset of GEO database. Therefore, gene expression profile analysis can better understand the genetic, cellular, and molecular changes that occur during the formation of corneal scar, which will provide an opportunity to design treatment options, selectively regulate the key stages of healing process, and thus produce scars closer to normal corneal structure [5].

In the present study, we downloaded an original microarray data set GSE6676 from the NCBI Gene Expression Comprehensive Database (NCBI-GEO) and analyzed to obtain DEGs between mice that have the overexpression of TGF beta and wildtype litter mate to the mice that have the overexpression of TGF beta. Scar formation of the cornea is critically modulated by the expression of transforming growth factor-beta (TGF-beta), TGF beta can induce the transformation of corneal keratocytes into myofibroblasts, which is the main cause of corneal fibrosis or scar formation [10]. Subsequently, in order to identify the related genes, pathways, and existing molecular mechanisms, we carried out Gene Ontology (GO), Kyoto Encyclopedia of Genes and Genomes (KEGG) enrichment pathway analyses and protein-protein interaction (PPI) network analyses. In summary, the mechanism of corneal scar formation was studied by bioinformatics, which provided potential biomarker candidates for clinical treatment and drug target discovery.

## 2. Materials and Methods

### 2.1. Microarray Data

GEO (http://www.ncbi.nlm.nih.gov/geo) [11] is a public functional genomics data repository of high throughout gene expression data, chips, and microarrays. One gene expression dataset [GSE6676] was downloaded from GEO (GPL1261 [Mouse430_2] Affymetrix Mouse Genome 430 2.0 Array). According to the annotation information in the platform, the probes are transformed into corresponding gene symbols. The GSE6676 data set contained 8 samples, including 4 corneas from wildtype mice samples and 4 corneas under the influence of high doses of TGF-beta samples.

### 2.2. Identification of Differentially Expressed Genes (DEGs)

The GEO2R (http://www.ncbi.nlm.nih.gov/geo/geo2r/) is used to identify differentially expressed genes (DEGs). GEO2R is based on *R* that comes with the GEO databases. Differentially expressed genes (DEGs) were determined by |logFC| of no less than 1 and *t*-tests with *P* < 0.05.

### 2.3. GO Enrichment and KEGG Pathway Analysis of the DEGs

The Database for Annotation, Visualization, and Integrated Discovery (DAVID; david. abcc. ncifcrf. Gov) software was used to interpret functions of extensive genes obtained from previous genome studies [12]. The Gene Ontology database (GO; http://www.geneontology.org) contained structured ontologies or vocabularies that depict basic characteristics of genes and gene products [13]. The Kyoto Encyclopedia of Genes and Genomes database (KEGG; http://www.genome.jp/kegg/) synthesizes information of biological systems from genomic, chemical, and systemic functional aspects [14]. Using the DAVID software, functional and pathway enrichment analyses were conducted separately, for upregulated and downregulated genes. *P* < 0.05 was considered to indicate a statistically significant difference for the screening of significant GO terms and KEGG pathways.

### 2.4. PPI Network Construction and Module Analysis

PPI networks are predicted by searching tools for interacting genes (STRING; (http://string-db.org)) (version 10.0) [15] online database. Analyzing the functional interactions between proteins may provide insights into the mechanisms of generation or development of diseases. In the present study, PPI network of DEGs was constructed using STRING database, and an interaction with a combined score >0.4 was considered statistically significant. Cytoscape (version 3.4.0) is an open source bioinformatics software platform for visualizing molecular interaction networks [16]. Cytoscape Plug-in Molecular Complex Detection (MCode) (Version 1.4.2) is an application for clustering a given network based on topology to discover densely connected regions [17]. The PPI network is drawn by Cytoscape, and the most important module is identified by MCODE. The criteria for selection were as follows: MCODE scores >5, degree cut-off = 2, node score cut-off = 0.2, Max depth = 100, and k-score = 2. Subsequently, the KEGG and GO analyses for genes in this module were performed using DAVID.

### 2.5. Hub Genes Selection

The hub genes were selected using the cytoHubba plugin, a Cytoscape plugin, was used to determine the hub proteins or genes in the PPI network. We used five methods (degree, Maximum Neighborhood Component (MNC), Radiality centrality, Stress centrality, Closeness centrality) to sequence and evaluate central genes [18].

## 3. Results

### 3.1. Identification of Differentially Expressed Genes (DEGs)

**Figure 1:**
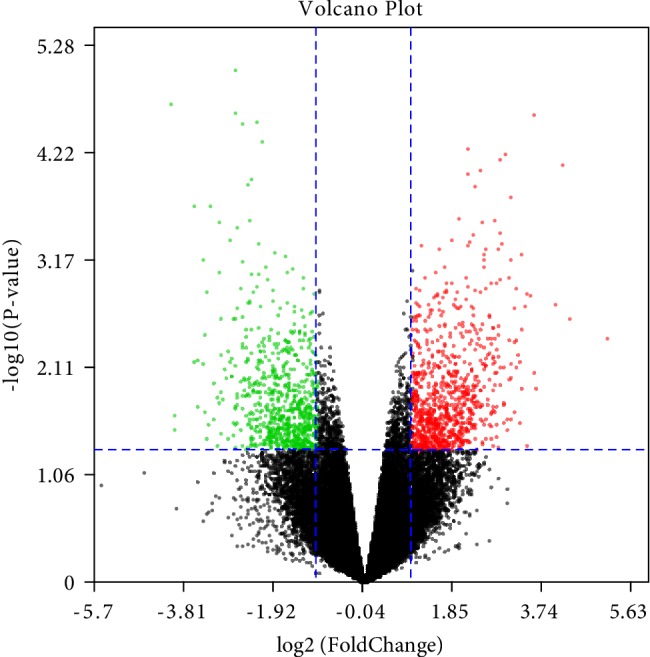
The volcano plot of all DEGs. Red represents up-regulated DEGs with log2FC >1 and *P* < 0.05. Green represents down-regulated DEGs with log2FC <−1 and *P* < 0.05. FC, fold-change; DEGs, differentially expressed genes.

**Figure 2:**
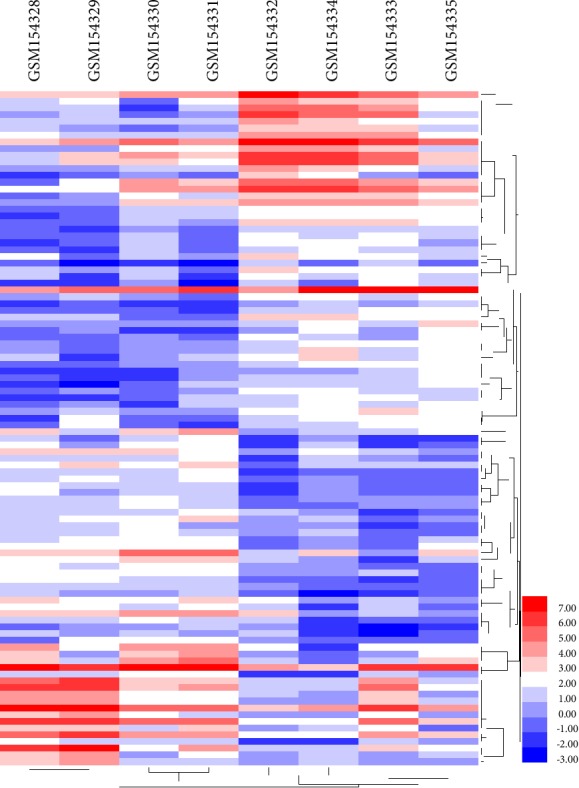
The heat map of the top 50 up-regulated genes and the top 50 down-regulated genes, blue indicates a relatively low expression and red indicates a relatively high expression.

We downloaded gene expression profile GSE6676 from the GEO database, and use the GEO2R method to identify DEGs in the corneas under the influence of high doses of TGF-beta samples compared with the corneas from wildtype mice samples. Based on the initial data, we included the inclusion criteria: *P* < 0.05 and |logFC| ≥ 1.0. A total of 1377 differentially expressed genes were identified, of which 776 were up-regulated and 601 were down-regulated between the corneas under the influence of high doses of TGF-beta samples compared with the corneas from wildtype mice samples. At the same time, the volcano plot of all DEGs and the heat map of the top 50 up-regulated genes and the top 50 down-regulated genes are shown in Figures 1 and 2, respectively.

### 3.2. GO Term Enrichment Analysis

**Figure 3:**
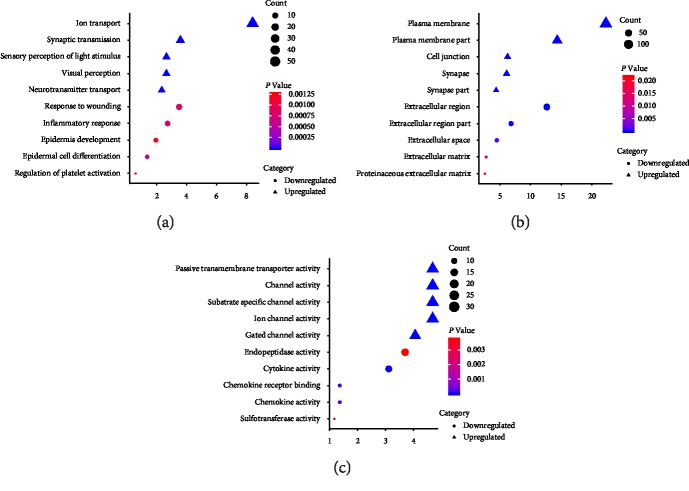
The Gene Ontology (GO) analysis of upregulated and downregulated differentially expressed genes.

Then, the online software DAVID was used to functionally categorize these 1377 significant DEGs. As shown in Figure 3, the top 5 enrichment analyses are shown for each part of gene ontology (GO) analysis. For biological process (BP) enrichment analysis, the results showed that the upregulated genes significantly took part in the ion transport (GO:0006811), synaptic transmission (GO:0007268), visual perception (GO:0007601), sensory perception of light stimulus (GO:0050953), and neurotransmitter transport (GO:0006836). The down-regulated genes were significantly involved in epidermal cell differentiation (GO:0009913), inflammatory response (GO:0006954), response to wounding (GO:0009611), epidermis development (GO:0008544), regulation of platelet activation (GO:0010543) as shown in Figure 3(a). For cell component (CC) enrichment analysis, the present study showed that the upregulated genes were mainly involved in synapse (GO:0045202), plasma membrane (GO:0005886), synapse part (GO:0044456), and plasma membrane part (GO:0044459), cell junction (GO:0030054). The Downregulated genes mainly revolved in extracellular region (GO:0005576), extracellular region part (GO:0044421), extracellular space (GO:0005615), extracellular matrix (GO:0031012), and proteinaceous extracellular matrix (GO:0005578) as shown in Figure 3(b). In addition, in the enrichment analysis of molecular function (MF), up-regulated genes are mainly enriched in ion channel activity (GO:0005216), substrate specific channel activity (GO:0022838), gated channel activity (GO:0022836), channel activity (GO:0015267), and passive transmembrane transporter activity (GO:0022803). For down-regulated genes, they mainly take part in cytokine activity (GO:0005125), chemokine activity (GO:0008009), chemokine receptor binding (GO:0042379), sulfotransferase activity (GO:0008146), and endopeptidase activity (GO:0004175) as shown in Figure 3(c).

### 3.3. KEGG Pathway Analysis

**Figure 4:**
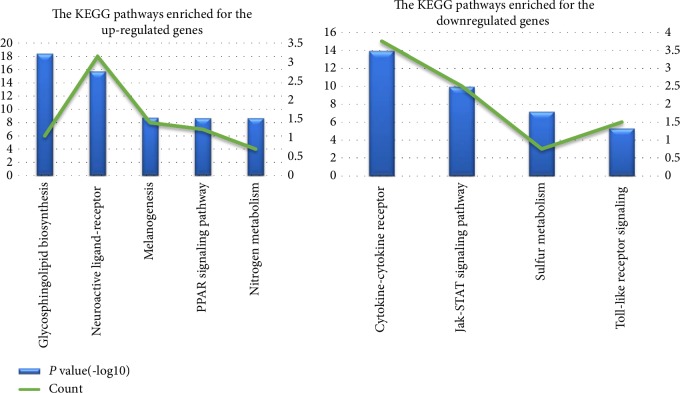
The KEGG pathway analysis of up-regulated and down-regulated differentially expressed genes.

KEGG pathway analysis showed that the up-regulated DEGs were mainly enriched in Glycosphingolipid biosynthesis (mmu00601), Neuroactive ligand-receptor interaction (mmu04080), Melanogenesis (mmu04916), PPAR signaling pathway (mmu03320), Nitrogen metabolism (mmu00910), while the down-regulated DEGs were mainly enriched in Cytokine-cytokine receptor interaction (mmu04060), Jak-STAT signaling pathway (mmu04630), Sulfur metabolism (mmu00920), Toll-like receptor signaling pathway (mmu04620). The KEGG pathway enrichment analysis results of up-regulated and down-regulated are shown in Figure 4.

### 3.4. Construction of PPI Network and Module Analysis

**Figure 5:**
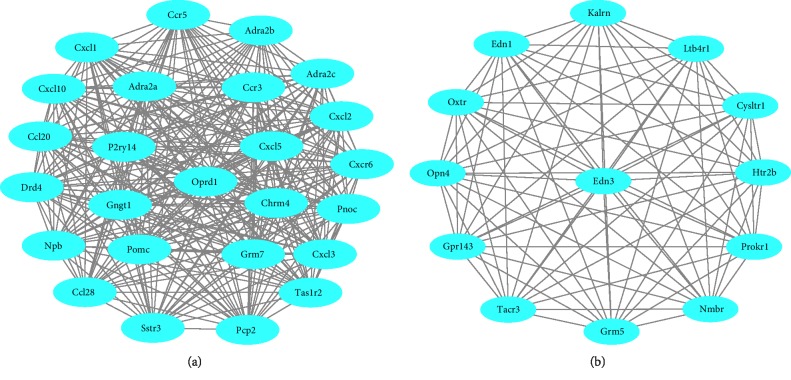
Top 2 modules from the protein-protein interaction network. (a) Module 1 and (b) module 2.

Based on the information in the STRING database, we used the plug-in MCODE in Cytoscape find 893 nodes and 3041 edges in the PPI network. Nodes represent DEGs and edges represent interactions between DEGs. Then, we use MCODE to filter the modules of PPI network. Therefore, two important modules are obtained from PPI network, as shown in Figure 5. Following the GO and KEGG pathway enrichment, analysis of DEGs in module 1 and module 2 were analyzed using DAVID. However the result shows that there were not go function enrichment and specific KEGG pathways in module 1, while the DEGs in module 2 was mostly distributed in the GO function enrichment. The module 2 most significantly took part in the molecular function (MF) as shown in Table1. For the KEGG pathway analysis, it was mainly distributed in Neuroactive ligand-receptor interaction and Calcium signaling pathway.

### 3.5. Hub Gene Selection

**Figure 6:**
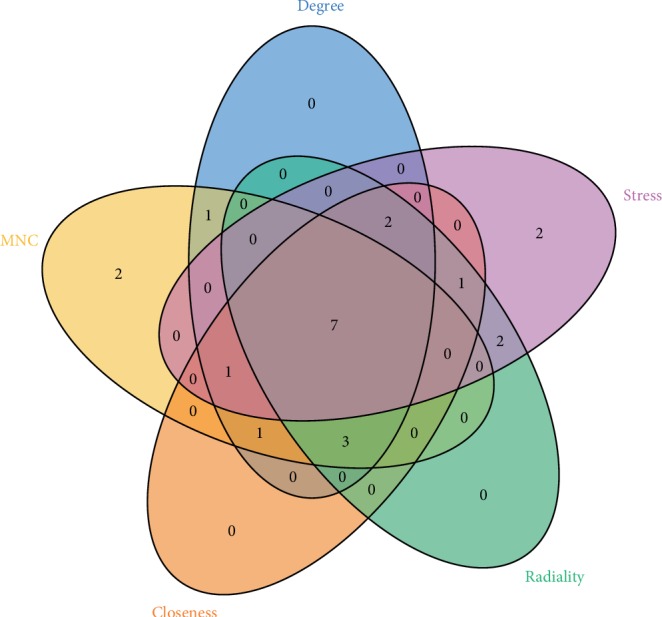
Seven hub genes were identified by overlapping the first 15 genes in the five classification methods of cytoHubba.

In the present study, we used cytoHubba to choose hub genes. According to the five classification methods in cytoHubba, the top 15 hub genes selected by these ranked methods in cytoHubba are shown in Table 2. Finally, seven central genes were identified by overlapping the first 15 genes, as shown in Figure 6. IL6 (interleukin-6) is the most excellent central genes based on five ranked methods. MMP9 (matrix metallopeptidase 9), CXCL10 (C-X-C motif chemokine ligand 10), MAPK8 (mitogen-activated protein kinase 8), TLR4 (toll-like receptor 4), HGF (hepatocyte growth factor), EDN1 (endothelin 1) were also selected as hub genes.

## 4. Discussion

The only widely accepted treatment for severe corneal scarring or clouding is the traditional techniques of corneal transplantation [19, 20]. The number of corneas required for these transplants, however, exceeds those available, leading to a shortage of donor tissues, especially in developing countries, which means that many patients can never undergo keratoplasty [20]. Therefore, it is very important to understand the precise molecular mechanism of corneal scar formation in order to formulate an effective diagnosis and look for alternative treatment strategies.

GEO2R is based on R software for analysis. GEO2R performs comparisons on original submitter-supplied processed data tables using the GEOquery and limma R packages from the Bioconductor project. Thus, GEO2R provides a simple interface that allows users to perform R statistical analysis without command line expertise. Both are equally accurate and many researchers have used GEO2R in the DEG analysis before. So we used GEO2R to analyze differentially expressed genes at our study.

In the present study, the gene expression profile of GSE6676 was downloaded and various bioinformatics analysis methods were performed to reveal the presence of differentially expressed molecules in an opaque cornea with blood vessels and lack of sensory nerves versus a normal cornea. Our study showed that there were 1377 differentially expressed genes in these two different clinical scenarios. The GO analyses showed that Upregulated DEGs mainly participated in cell component (CC), while down regulated DEGs mainly took part in both cell component (CC) and molecular function (MF). The upregulated genes were mainly enriched in Synapse, synapse part, plasma membrane, plasma membrane part, and ion transport. However the downregulated genes were mainly involved with extracellular region, extracellular region part, cytokine activity, chemokine activity, and chemokine receptor binding. Synapses and synapse part may be related to nerves in corneal wound healing. Corneal nerves are very important to maintain the protective reflexes (blinking and tearing) and to provide support to epithelial regeneration, through growth factors [21, 22]. Plasma membranes and ion transport may be due to a large amount of material exchange during corneal wound healing. Some study had shown that during the healing process of epithelial wounds, a large amount of extracellular matrix is needed to promote the migration of epithelial cells to cover the wounds [23]. More importantly, they play an important role in signaling cross-talk. Previous evidence suggests the presence and importance of intracellular signal crosstalk during epithelial healing after injury [2]. Previous studies have reported that extracellular region and extracellular region part play important roles in the cornea wound repair. For example, extracellular matrix (ECM) provides chemical and physical signals to keratocytes, thus affecting their differentiation to fibroblasts and/or myofibroblasts, and plays an important role in the formation of corneal scar. It also provides a matrix through which they migrate during wound repair [24–26]. In addition, recent studies have shown that cytokines and chemokines are important regulators of wound healing-related cell growth, proliferation, migration, differentiation, adhesion, ECM deposition, and protease regulation. Corneal cells express a variety of growth factors and cytokines that have special effects on epithelial cells. It is activated during corneal wound healing and plays a major role in corneal inflammation and angiogenesis [27]. THE KEGG pathway analysis also indicated that the DEGs were mainly enriched in Neuroactive ligand-receptor interaction and Cytokine-cytokine receptor interaction, this is consistent with our previous results of functional enrichment analysis. Ji et al. [28] showed that the cytokine-cytokine-receptor interaction pathway is an important way to participate in miR-296 gene-related inflammation in cornea of alkali burned mice.

The PPI network of DEGS is constructed by strings and visualized by cytoscape. Five significant modules (score ≥5) were identified by MCODE, then the GO and KEGG pathway enrichment analysis of DEGs in this most significant module was analyzed using DAVID. Our study shows that no specific GO and KEGG pathways were found in module 1. Module 2 most significantly took part in G-protein coupled receptor protein signaling pathway, cell surface receptor linked signal transduction, peptide receptor activity, G-protein coupled, peptide binding, and neuropeptide receptor activity. G-protein coupled receptor protein signaling pathway, peptide receptor activity, and G-protein coupling played important roles in Lysophosphatidic Acid Promoting Corneal Epithelial Wound Healing. LPA accelerates corneal epithelial wound healing by inducing the ability of autocrine HB-EGF signal. G protein-coupled receptor ligands can be used as stimulants to activate LPA pathway and epidermal growth factor receptor to meet the pathophysiological challenges of human corneal epithelial cells [29]. Previous study showed that some cell surface receptor linked signal transduction plays a major role in the pathogenesis of corneal neovascularization, such as Vascular Endothelial Growth Factors (VEGFs). Vascular endothelial cell proliferation can be promoted by allogeneic T cells from corneal transplantation host by using VEGF-A signaling. Vascular endothelial growth factor is one of the important factors affecting corneal neovascularization. The role of vascular endothelial growth factor in corneal neovascularization has been confirmed. The inhibitory effect of vascular endothelial growth factor is currently being studied as a treatment for corneal neovascularization [30, 31]. KEGG pathways in module 2 were mainly involved in Neuroactive ligand-receptor interaction and Calcium signaling pathway. The Neuroactive ligand-receptor interaction was also found in KEGG pathway analysis of the up-regulated DEGs. Neuroactive ligands can transmit important signals to maintain corneal reflex and recovery of nervous function [21]. Some study showed that early reactions after corneal epithelial injury include the release of nucleotides, the transmission of calcium waves from the wound, and the promotion of migration to reconstruct the cytoskeleton rearrangement of the epithelial barrier. So calcium signaling pathway promotes migration to reestablish the epithelial barrier [32]. In addition, calcium blocker diltiazem is to be used for therapeutic control of pain following corneal injury or surgery because it can retain blink reflex and promote wound healing [33]. Therefore, an in-depth understanding of these pathways is helpful to elucidate the key mechanism of corneal wound healing.

Moreover, we analyzed the PPI network of the DEGs and seven hub genes were identified by five sequencing methods in cytoHubba: IL6 (interleukin-6), MMP9 (matrix metallopeptidase 9), CXCL10 (C-X-C motif chemokine ligand 10), MAPK8 (mitogen-activated protein kinase 8), TLR4 (toll-like receptor 4), HGF (hepatocyte growth factor), EDN1 (endothelin 1). IL6, the most outstanding hub gene, which had been reported to play an important role in ocular surface immune defense and decreases the severity and susceptibility of contact lens–related keratitis [34]. However Di Girolamo illustrated IL6 that are induced by UV radiation may play a key role in initiating blood vessel formation, cellular proliferation, tissue invasion, and inflammation [35]. Ghasemi also found IL-6 plays and modulates many important roles in ocular inflammation and angiogenesis in the cornea and it can result in unwanted neovascularization or exacerbation of inflammation, leading to the destruction and damage of delicate eye factors [36]. In addition, IL-6 can stimulate neutrophil degranulation, causing localized tissue damage with loss of corneal transparency and visual impairment [37]. In a word, IL-6 plays a dual role in the cornea. MMP9 (matrix metallopeptidase 9), as one of imperative matrix metalloproteinase, synthesized by corneal epithelial cells and stromal cells, have long been suspected of having a significant role in keratoconus [38]. Matrix metalloproteinases (MMPs) affect cell migration by degradation of extracellular matrix or by altering cell adhesion properties, and increase during corneal wound healing. Previous studies have shown that Galectin-3-induced MMP-9 promotes corneal cell movement and wound healing by destroying cell contact, while knockout mice show delayed skin wound healing in vivo [39, 40]. Groblewska et al. reported that MMP-9 are able to degrade collagen type IV from the basement membrane and ECM, a process that is related to tumor progression, metastasis, growth, and angiogenesis [41]. Du et al. had reported that blockade of MMP -9 could inhibit corneal lymphangiogenesis [42]. A better understanding of this factor may provide novel therapies for corneal transplant rejection and other lymphatic disorders. CXCL10 (C-X-C motif chemokine ligand 10) as a member of the interferon-inducible tripeptide motif Glu-Leu-Arg-negative (ELR-) CXC chemokines, is a fibrotic and vascular inhibitory chemokine produced by macrophages, endothelial cells, and fibroblasts. It is reported to be involved in the natural defense of cornea against microbial infection. Interferon regulatory factors (IRF1) play a role in corneal innate immune response by regulating the expression of CXCL10. IFN*γ*-producing NK cells enhance the epithelial expression of IRF1 and CXCL10, thus contributing to the corneal natural defense against Pseudomonas aeruginosa infection [43]. Gao et al. reported that CXCL10 in inflammatory cornea may be an endogenous factor, which can prevent angiogenesis and control vascular regression. In addition, CXCL10 can be locally applied to the eye surface by derivative peptide or AAV-CXCL10 to reduce or prevent corneal angiogenesis associated with chemical damage and graft rejection, which is related to corneal lymphangiogenesis [44]. MAPK8, also known as JNK1, is a member family of mitogen-activated protein kinases (MAPK), belonging to conserved serine and threonine protein kinases. Some studies have shown that c-Jun Nterminal kinase (JNK) 1 and 2 play an important role in the formation of corneal scar. The increase of JNK 1/2 level can block the expression of connective tissue growth factor gene induced by TGF-beta 1 and subsequent cell proliferation, migration, and collagen synthesis [45, 46]. Previous studies have shown that JNK1/2 participates in the expression of CTGF induced by TGF-beta 1. JNK1/2 pathway inhibitor SP600125 plays a role in the inhibition of CTGF expression. Therefore, the study and identification of JNK1/2 pathway may help to provide a new strategy to reduce corneal scar formation [46]. TLR4 (Toll-like receptor 4) plays an important role in corneal epithelial wound healing. Eslani et al. show that Functional TLR 4 expression is induced after corneal epithelial injury, then Toll-like receptor 4 signal promotes epithelial repair by inducing cell migration, proliferation, and regulates the production of inflammatory cytokines in damaged corneal epithelium [47]. The wound closure rate of human corneal epithelial cells (HCEC) treated with lipopolysaccharide (LPS) increased significantly. In these cells, TLR4 mediates the increase of cytokines such as IL-6, TNF-a, and CXCL8/IL-8, and the phosphorylation of ERK1/2 and p38 MAP kinases [2]. Therefore, TLR4 contributes to inflammatory response and epithelial cell migration and proliferation. Therefore, the regulation of TLR4 signal may provide a new treatment for corneal epithelial wound healing disorder. Hepatocyte growth factor (HGF) and its receptor protein was found in cornea [48]. Gupta et al. show that HGF plays an important role in corneal repair through c-met receptor tyrosine kinase [49]. HGF exists in three main cell types of human cornea, including epithelial cells, stromal cells, and endothelial cells. Corneal epithelial injury can up-regulate the expression of HGF in corneal cells, and then regulate corneal wound healing through secretion, autocrine, and paracrine ways [50]. Moreover Gupta et al. reported that Localized topical BMP7 + HGF gene therapy can treat improving transparency and corneal fibrosis in vivo, and reduce overhealing and selective apoptosis of myofibroblasts [49]. EDN1 is a key protein of non-neuronal autoregulation of retinal blood flow, which can cause vasoconstriction and vascular tension, and enhance endothelial production of vasodilators such as nitric oxide and prostacyclin [51]. The interaction between vascular endothelial growth factor (VEGF) and EDN1 may play an important role in the simultaneous proliferation of vascular wall endothelial cells and smooth muscle cells. Jinzi Zhou had reported elevated EDN1 in vitreous of patients with proliferative diabetic retinopathy (PDR) [52]. However, there was no report shown that whether EDN1 plays a role in corneal wound healing or not.

However, the limitation of our study is that there is no dataset of humans related to corneal scar in the GEO database. We only found the dataset of mouse. Although it can not be directly adapted to human beings, it will be of great help to our next research. In addition, based on our analysis, we can infer the potential mechanism of Hub gene in corneal scar, but it needs further molecular biological experiments to verify and confirm the potential mechanism of hub genes in corneal scar formation.

## 5. Conclusion

In conclusion, 1 377 DEGs, 7 hub genes, and 2 important network modules were identified. Seven hub genes IL6, MMP9, CXCL10, MAPK8, TLR4, HGF, and EDN1 and their signaling pathways, identified in this research, warrant further studies in order to explore their application in the clinical setting. However, molecular biological experiments are required to confirm the function of the identified genes in the formation of corneal scar.

## Figures and Tables

**Table 1:** The top 10 Gene Ontology (GO) functions and Kyoto Encyclopedia of Genes and Genomes (KEGG) pathways enriched for the genes involved in module 2.

Description	*P* Value	Count	Gene symbol
GO:0007186 G-protein coupled receptor protein signaling pathway (BP)	1.25E-07	11	GRM5, LTB4R1, CYSLTR1, TACR3, EDN1, OXTR, PROKR1, GPR143, NMBR, HTR2B, OPN4
GO:0007166 cell surface receptor linked signal transduction (BP)	1.97E-06	11	GRM5, LTB4R1, CYSLTR1, TACR3, EDN1, OXTR, PROKR1, GPR143, NMBR, HTR2B, OPN4
GO:0008528 peptide receptor activity, G-protein coupled (MF)	6.35E-05	4	TACR3, OXTR, PROKR1, NMBR
GO:0042277 peptide binding (MF)	1.93E-04	4	TACR3, OXTR, PROKR1, NMBR
GO:0008188 neuropeptide receptor activity (MF)	3.53E-04	3	TACR3, PROKR1, NMBR
GO:0042165 neurotransmitter binding (MF)	0.001843	3	TACR3, PROKR1, NMBR
GO:0004974 leukotriene receptor activity (MF)	0.003007	2	LTB4R1, CYSLTR1
GO:0003013 circulatory system process (BP)	0.004138	3	EDN3, EDN1, OXTR
GO:0005886 plasma membrane (CC)	0.009375	8	GRM5, LTB4R1, CYSLTR1, TACR3, OXTR, PROKR1, NMBR, HTR2B
GO:0042310 vasoconstriction (BP)	0.011425	2	EDN3, EDN1
mmu04080: Neuroactive ligand-receptor interaction	5.51E-08	7	GRM5, LTB4R1, CYSLTR1, TACR3, OXTR, NMBR, HTR2B
mmu04020: Calcium signaling pathway	3.85E-05	5	GRM5, CYSLTR1, TACR3, OXTR, HTR2B

**Table 2:** The top 15 hub genes rank in cytoHubba.

Degree	Stress	MNC	Radiality	Closeness
IL6	IL6	IL6	IL6	IL6
GNGT1	UBC	CXCL1	MAPK8	MAPK8
CXCL10	MAPK8	CXCL10	TLR4	TLR4
TLR4	TLR4	GNGT1	INS2	MMP9
CXCL1	GNGT1	MMP9	HGF	CXCL10
MMP9	POMC	CXCL2	MMP9	INS2
MAPK8	INS2	TLR4	POMC	POMC
CXCL2	SLCLA2	CCR5	CXCL10	HGF
CCR5	HGF	MAPK8	EDN1	CXCL1
UBC	SYP	CCL20	SLCLA2	EDN1
POMC	CXCL10	CXCL5	CXCL1	CXCL2
CCL20	PVALB	EDN1	UBC	CCR5
CXCL5	EDN1	HGF	CCR5	UBC
HGF	RAB3A	CXCL3	CCL20	CCL20
EDN1	MMP9	CCR3	SYP	GNGT1

## Data Availability

All data generated or analyzed during this study are included in this published article.
